# Comparison of Microendoscopic Laminotomy (MEL) Versus Spinous Process-Splitting Laminotomy (SPSL) for Multi Segmental Lumbar Spinal Stenosis

**DOI:** 10.7759/cureus.22067

**Published:** 2022-02-09

**Authors:** Ryunosuke Oyama, Takeshi Arizono, Akihiko Inokuchi, Ryuta Imamura, Takahiro Hamada, Hirofumi Bekki

**Affiliations:** 1 Orthopedic Surgery, Kyushu Central Hospital of the Mutual Aid Association of Public School Teachers, Fukuoka, JPN; 2 Orthopaedic Surgery, Kyushu Central Hospital of the Mutual Aid Association of Public School Teachers, Fukuoka, JPN

**Keywords:** minimally invasive surgery, spinous process-splitting laminotomy, microendoscopic laminotomy, multi segmental, lumbar spinal stenosis

## Abstract

Aims

This study was aimed to compare the perioperative and postoperative outcomes of patients who underwent posterior decompression for multi-segmental lumbar spinal stenosis by microendoscopic laminotomy (MEL) versus spinous process-splitting laminotomy (SPSL) retrospectively.

Methods

We retrospectively reviewed 73 consecutive patients who underwent two or three levels MEL (n=51) or SPSL (n=22) for lumbar spinal stenosis between 2012 and 2018. The perioperative outcomes were operative time, intraoperative blood loss, length of postoperative hospital stay, complications, and reoperation rate. The postoperative outcomes were evaluated using a visual analog scale (VAS) and the Japanese Orthopaedic Association Back Pain Evaluation Questionnaire (JOABPEQ) scores at one year postoperatively.

Results

The mean follow-up time was 26.6 months in MEL and 35.6 months in SPSL. The mean operative time was significantly longer in MEL than SPSL (two levels, 183.6 ± 43.2 versus 134.8 ± 26.7 min, respectively; three levels: 241.6 ± 47.8 versus 179.9 ± 28.8 min, respectively). MEL's mean postoperative hospital stay was significantly shorter than SPSL (12.3 ± 5.9 versus 15.5 ± 7.2 days, respectively). There was no significant difference in the mean intraoperative blood loss, complication rate, reoperation rate, and postoperative outcomes between the two groups.

Conclusions

This study suggests that both techniques are effective in treating multi-segmental lumbar spinal stenosis. There was no significant difference between the two procedures in intraoperative blood loss (IBL), complications rate, reoperation rate, or improvement in VAS and Japanese Orthopaedic Association Back Pain Evaluation Questionnaire (JOABPEQ) scores. MEL had an advantage in the postoperative hospital stay.

## Introduction

In treating lumbar spinal stenosis, minimally invasive surgery has been increasingly utilized to preserve the paraspinal muscle attachments and the posterior ligamentous complex, which are associated with postoperative back muscle atrophy and lumbar instability, and low back pain [1,2]. Microendoscopic laminotomy (MEL) was introduced by Foley and Smith [3] in 1997 and was initially performed using Minimal Exposure Tubular Retractor MicroDiscectomy (METRx-MD) system, in which a tubular retractor was used. In 2002, Palmer et al. [4] described the performance of unilateral laminectomy for bilateral decompression using a micro endoscope for lumbar spinal stenosis. MEL for lumbar spinal stenosis has been recognized to have several advantages, such as smaller incisions, less blood loss, and a shorter hospital stay than conventional open laminectomy [5-9].

Another technique, spinous process-splitting laminotomy (SPSL), was developed by Watanabe et al. [10]. In SPSL, the spinous process is vertically split, preserving its paravertebral muscular and ligamentous attachments. SPSL for lumbar spinal stenosis has also demonstrated better clinical results than conventional open laminectomy [11-13].

Although favorable clinical results were reported previously for MEL and SPSL, few studies have compared these two procedures in multi-segmental lumbar spinal stenosis. This study examined the perioperative and postoperative outcomes of patients who underwent posterior decompression for multi-segmental lumbar spinal stenosis using MEL versus SPSL.

## Materials and methods

Patient selection

We performed a retrospective electronic medical record review of all consecutive patients who underwent posterior decompression surgery at our institution between January 2012 and December 2018. A total of 608 patients were initially identified. The inclusion criteria were posterior decompression surgery of two or three levels with at least one year of follow-up. We excluded patients who had a history of lumbar spinal surgery in other hospitals; had undergone concomitant thoracic laminectomy, instrumented fusion, or kyphoplasty; had degenerative spondylolisthesis or scoliosis (Cobb angle >10°); and whose follow-up data were missing. Four spine surgeons performed all procedures, and surgeons selected the surgical procedure according to their preference. All patients were scheduled to visit our hospital for periodic medical examinations. Our institutional review board approved this study and written informed consent was obtained from all participants.

Clinical data

We collected the following data regarding the patients' baseline and operative characteristics from their medical records: age, sex, body mass index, American Society of Anaesthesiologists physical status (ASA-PS), decompressed disc level, and rate of concomitant discectomy. The ASA-PS classification system is a category classification system for assessing patients' surgical risk and comorbidities before surgery: Class 1, healthy; Class 2, mild systemic disease; and Class 3, severe systemic disease.

The perioperative outcomes were operative time, intraoperative blood loss (IBL), length of postoperative hospital stay, complications, and reoperation rate during the follow-up period. The IBL was determined based on blood in the swabs and suction canisters. If the blood loss was too low to be measured, it was recorded as 1 ml. The length of the postoperative hospital stay was measured in days from the first postoperative day until discharge. The patients were discharged when they could walk more than 50 m continuously.

The postoperative outcomes were evaluated using a visual analog scale (VAS; 0-10 cm) and the Japanese Orthopaedic Association Back Pain Evaluation Questionnaire (JOABPEQ). JOABPEQ consists of 24 questions classified into five categories: low back pain, lumbar function, walking ability, social life function, and mental health, and the five scores range from 0 to 100 points with a higher value indicating a better condition [14]. These scores were obtained before surgery and at one year postoperatively, and calculate the difference in each score.

Surgical technique

All procedures were performed with the patients under general anesthesia. The patients were placed in the prone position on a Jackson table.

MEL approach

**Figure 1:**
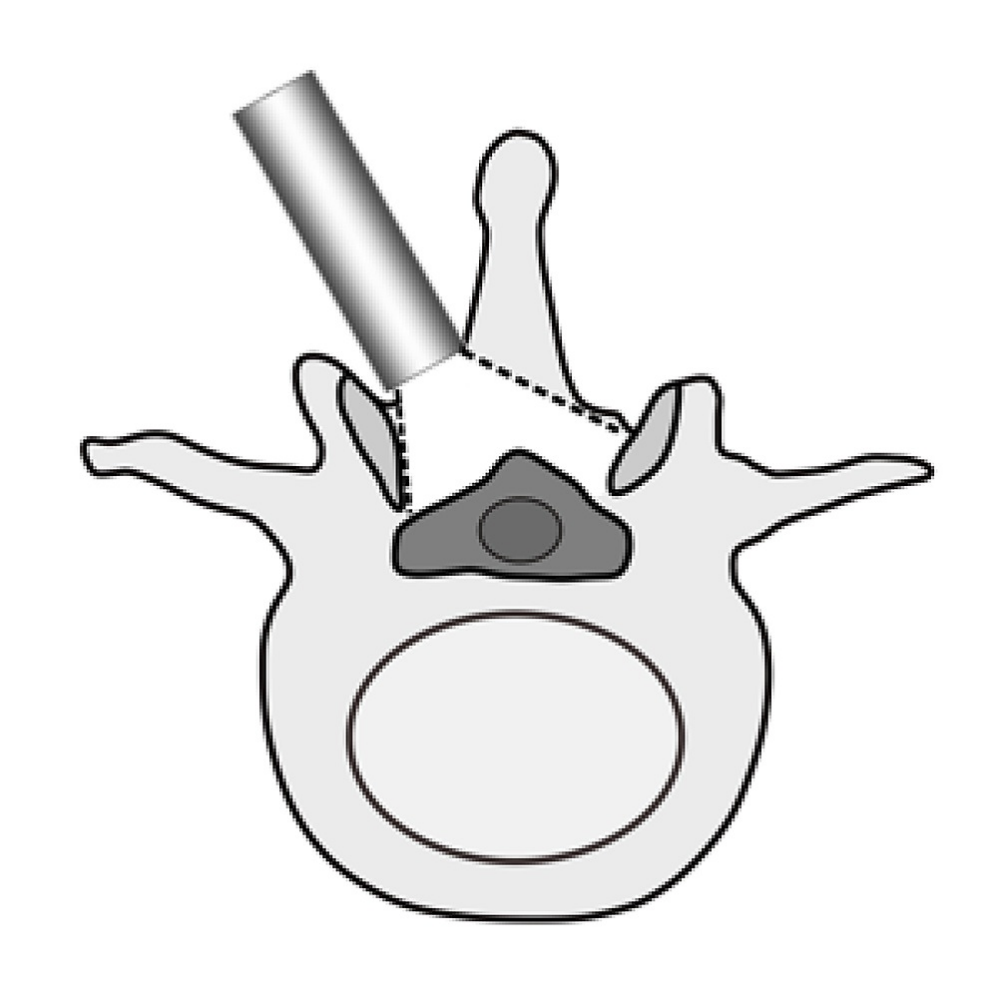
Diagram for MEL techniques A skin incision was made 5 mm lateral to the spinous process, and a tubular retractor was placed. Unilateral laminotomy for bilateral decompression was performed in the operative field. MEL, Microendoscopic laminotomy.

Unilateral laminotomy for bilateral decompression with tubular retractors was performed [4]. A skin incision was made 5 mm lateral to the spinous process, the muscle was sequentially dilated, and a tubular retractor was placed. Laminotomy was initially performed from the inferior edge of the lamina to the transition between the spinous process and lamina. The tubular retractor was tilted inward, and laminotomy of the base of the spinous process and the contralateral lamina was then performed. After securing the working space, facetectomy was performed from the approach side to the contralateral side using an air drill or Kerrison punch. Decompression was performed after resectioning the ligamentum flavum and exposure of the dural sac and nerve root (Figure 1). After decompression, the surgical site was irrigated with physiological saline, and a suction drain was placed in the epidural space. The tubular retractor was withdrawn, and the incision was closed.

SPSL approach

**Figure 2:**
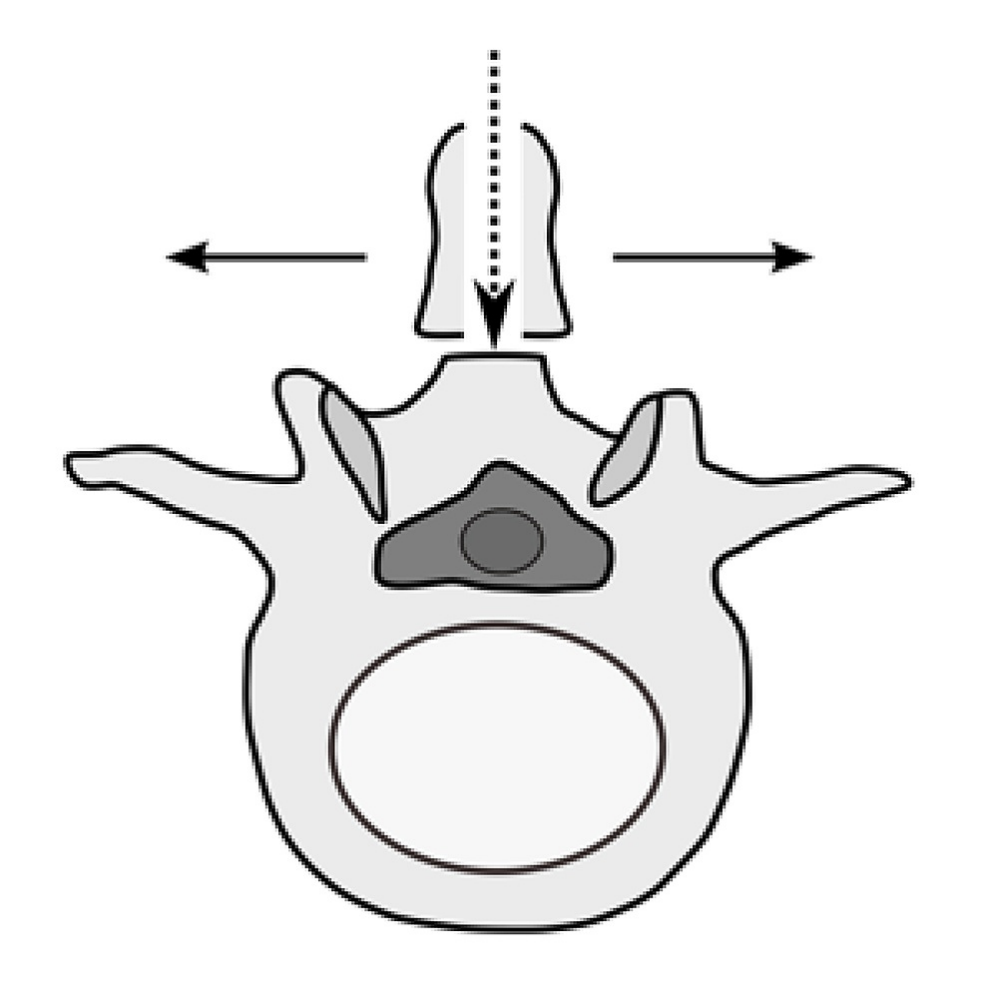
Diagram for SPSL techniques A midline skin incision was made over the spinous processes, and the spinous process split longitudinally in the middle, divided at the base, and detached from the lamina, leaving the bilateral paraspinal muscles attached to each split spinous process. Decompression was performed in the operative field. SPSL, spinous process-splitting laminotomy.

The SPSL procedure was performed according to the method described by Watanabe et al. [10]. A midline skin incision was made over the spinous processes to expose the tip. The spinous process was split longitudinally in the middle, divided at the base, and detached from the lamina, leaving the bilateral paraspinal muscles attached to each split spinous process. The muscle attached to the lamina was dissected using a Cobb elevator. Laminectomy was followed by decompression after resection of the flavum (Figure 2). The surgical field was irrigated, and the split spinous processes and interspinous or supraspinous ligaments were sutured; finally, a suction drain was placed. The performance of concomitant discectomy, when required, was left to the discretion of the surgeon in both techniques.

Data analysis

All statistical analyses were performed with R (version 4.1.2). The Mann-Whitney U-test was used to compare continuous variables between the two groups, and the chi-squared test was used to compare categorical variables. A p-value of <0.05 was considered statistically significant.

## Results

**Figure 3:**
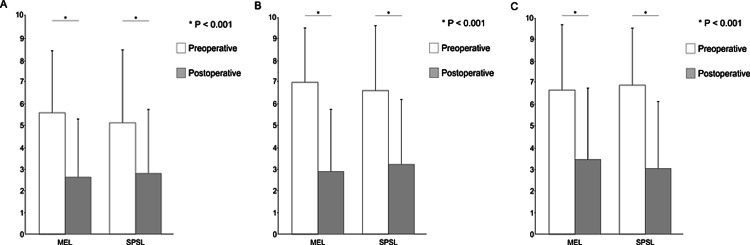
Improvement of VAS score The mean preoperative (white) and postoperative (grey) scores are graphed. A, B, C are low back pain, leg pain, and leg numbness score respectively. There was no differences in the improvement of VAS sores between the two groups. MEL, Microendoscopic laminotomy; SPSL, spinous process-splitting laminotomy.

**Figure 4:**
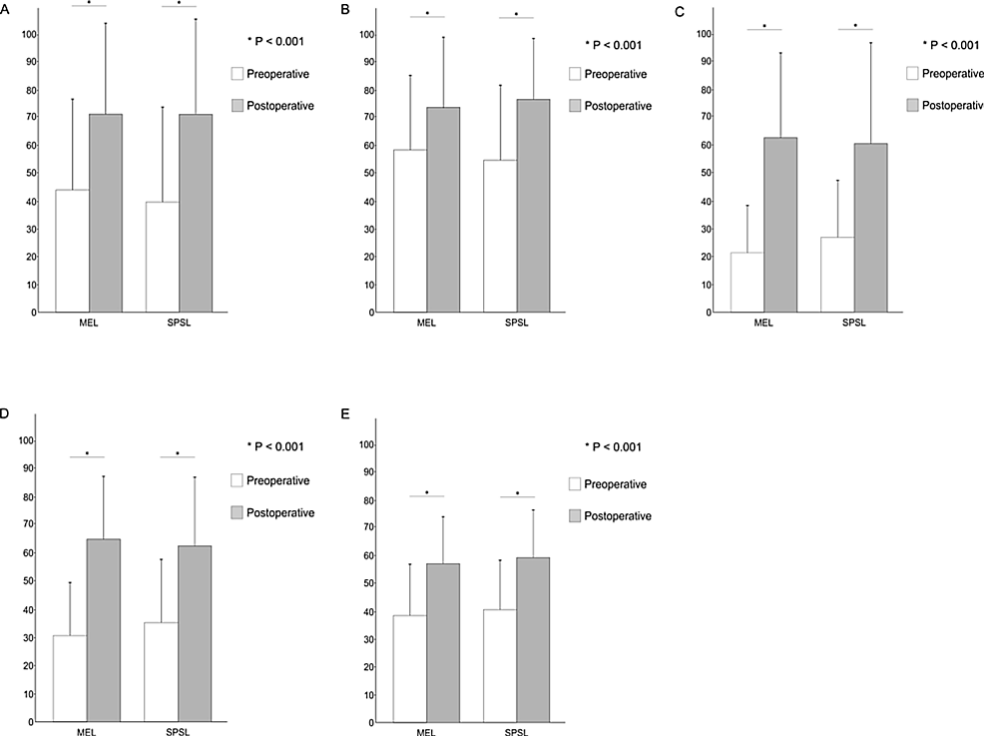
Improvement of JOABPEQ scores The mean preoperative (white) and postoperative (grey) scores are graphed. A, B, C, D, E are low back pain, lumbar function, walking ability, social function, and mental health score respectively. There was no differences in the improvement of JOABPEQ sores between the two groups. MEL, Microendoscopic laminotomy; SPSL, spinous process-splitting laminotomy.Improvement of JOABPEQ score. Graph shows the mean ± SD values for both MEL (white) and SPSL (grey) groups.

MEL and SPSL have been increasingly utilized to preserve the paraspinal muscle attachments and the posterior ligamentous complex, which are associated with postoperative back muscle atrophy, lumbar instability, and low back pain. This study involved 73 patients (MEL, n = 51; SPSL, n = 22) who met the inclusion criteria. The patients' characteristics are presented, the preoperative VAS and JOABPEQ scores and the perioperative outcomes are presented in tables 1-3. The mean operative time was significantly longer in the MEL than SPSL group for two levels of decompression (183.6 ± 43.2 versus 134.8 ± 26.7 min, respectively; p < 0.001) and three levels of decompression (183.6 ± 43.2 versus 134.8 ± 26.7 min, respectively; p = 0.011). The mean length of the postoperative hospital stay was significantly shorter in the MEL than SPSL (12.3 ± 5.9 versus 15.5 ± 7.2 days, respectively; p = 0.004). There was no significant difference in the mean IBL, complications, and reoperation rate between the two groups. Two patients in MEL underwent reoperations. One underwent discectomy at another disc level 21 months later, and another underwent discectomy at the same level 24 months later. Furthermore, two patients in the SPSL group underwent reoperations. One underwent posterior lumbar interbody fusion at the same vertebral level 72 months later, and another underwent MEL at the same level 75 months later. The postoperative outcomes are presented in table 4, figures 3-4. There was no significant difference in VAS and JOABPEQ scores between the two groups.

**Table 1:** Baseline and operative characteristics Abbreviation: MEL, microendoscopic laminotomy; SPSL, spinous process-splitting laminotomy; ASA-PS, American Society of Anaesthesiologists physical status; BMI, body mass index; SD, standard deviation

Surgical techniques		p-value
	MEL (n=51)	SPSL (n=22)	
Age, mean ± SD	69.1 ± 10.0	66.2 ± 9.8	0.23
Follow-up (months), mean ± SD	26.6 ± 14.3	35.6 ± 20.6	0.002
Female sex, n (%)	10 (20)	3 (14)	0.74
BMI (kg/m²), mean ± SD	25.6 ± 3.9	25.2 ± 3.9	0.74
ASA-PS, n (%)			0.18
1	6 (12)	2 (9)	
2	39 (76)	13 (59)	
3	6 (12)	7 (32)	
Number of decompressed levels, n (%)			0.033
2 levels	43 (84)	13 (59)	
3 levels	8 (16)	9 (41)	
Number of each decompressed level, n			
L1/2	1	1	
L2/3	10	7	
L3/4	40	14	
L4/5	47	20	
L5/S1 (L6)	12	9	
Concomitant discectomy, n (%)	32 (63)	12 (72)	0.59

**Table 2:** Preoperative VAS and JOABPEQ scores Abbreviation: MEL, microendoscopic laminotomy; SPSL, spinous process-splitting laminotomy; JOABPEQ, Japanese Orthopaedic Association Back Pain Evaluation Questionnaire; VAS, visual analog scale

Surgical techniques		p value
	MEL (n=51)	SPSL (n=22)	
VAS score, mean ± SD			
Low back pain	5.7 ± 2.9	5.2 ± 3.4	0.55
Leg pain	7.0 ± 2.5	6.6 ± 3.0	0.56
Leg numbness	6.7 ± 3.0	6.9 ± 2.7	0.78
JOABPEQ score, mean ± SD			
Low back pain	44.0 ± 32.8	39.7 ± 34.7	0.45
Lumbar function	58.6 ± 27.0	54.9 ± 27.3	0.59
Walking ability	21.4 ± 17.2	27.3 ± 20.3	0.21
Social life function	30.8 ± 18.9	35.3 ± 22.7	0.38
Mental health	38.4 ± 18.4	40.6 ± 17.8	0.63

**Table 3:** Perioperative outcomes Abbreviation: MEL, microendoscopic laminotomy; SPSL, spinous process-splitting laminotomy

urgical techniques		p value
	MEL (n=51)	SPSL (n=22)	
Operative time (min), mean ± SD			
2 levels	183.6 ± 43.2	134.8 ± 26.7	< 0.001
3 levels	241.6 ± 47.8	179.9 ± 28.8	0.011
Intraoperative blood loss (ml), mean ± SD			
2 levels	28.9 ± 37.0	38.3 ± 31.6	0.12
3 levels	41.5 ± 33.2	89.9 ± 57.4	0.092
Postoperative hospital stay (days), mean ± SD	12.3 ± 5.9	15.5 ± 7.2	0.004
Complications, n (%)	12 (24)	2 (9)	0.20
Dural tear	5 (10)	1 (5)	0.45
Epidural hematoma	6 (12)	1 (5)	0.34
Pneumonia	1 (2)	0 (0)	0.51
Reoperation, n (%)	2 (4)	2 (9)	0.58

**Table 4:** Improvement in VAS and JOABPEQ scores Abbreviation: MEL, microendoscopic laminotomy; SPSL, spinous process-splitting laminotomy; JOABPEQ, Japanese Orthopaedic Association Back Pain Evaluation Questionnaire; VAS, visual analog scale; SD, standard deviation

Surgical techniques		p value
	MEL (n=51)	SPSL (n=22)	
VAS score, mean ± SD			
Low back pain	3.1 ± 3.0	2.4 ± 4.0	0.57
Leg pain	4.2 ± 3.5	3.4 ± 3.6	0.39
Leg numbness	3.2 ± 3.5	4.2 ± 3.8	0.18
JOABPEQ score, mean ± SD			
Low back pain	27.4 ± 43.9	31.6 ± 39.5	0.95
Lumbar function	15.0 ± 28.4	21.9 ± 32.0	0.38
Walking ability	41.6 ± 34.8	33.2 ± 39.1	0.55
Social life function	34.5 ± 28.9	27.2 ± 30.9	0.30
Mental health	19.1 ± 20.0	18.9 ± 24.7	0.64

## Discussion

In treating lumbar spinal stenosis, MEL and SPSL have been increasingly utilized in recent years to preserve the paraspinal muscle attachments and the posterior ligamentous complex. However, when it comes to multi-segmental lumbar spinal stenosis, it seems that the surgical procedure can be more invasive with more blood loss or longer operative time. This study compared the perioperative and clinical outcomes of the two procedures in multi-segmental lumbar spinal stenosis.

Regarding perioperative outcomes, the mean operative time was significantly longer in the MEL than SPSL group. Previous studies in which MEL was compared with conventional laminectomy for degenerative spinal stenosis showed no significant difference in the operative time [5-8]. This result may be associated with the difficulties of using a tubular retractor system with which surgeons are forced to operate in a smaller working space and with limited maneuverability of instruments [15].

In this study, the mean length of the postoperative hospital stay was 3.2 days shorter in MEL than SPSL, with a statistically significant difference. This result is nearly identical to that reported in other studies in Japan [12,16]. Many independent variables can influence the length of the hospital stay, such as age, comorbidities, intraoperative factors, and postoperative complications. Gruskay et al. [17] performed multivariate analysis and found that older patients with widespread systemic disease tended to stay in the hospital longer after posterior lumbar spine surgery. Our results suggest that MEL can result in earlier mobilization even for elderly patients, which may potentially lower hospital resource utilization in acute care.

Several potential disadvantages have been previously raised with MEL for lumbar stenosis. First, because of limited visualization of critical structures, such as dura and nerve roots, some have claimed that this may be responsible for higher rates of accidental durotomy [18]. Second, MEL is a complex, technically challenging procedure requiring considerable experience to decompress neural structures adequately. The unilateral tubular technique has minimal vision and physical space to manipulate surgical equipment, which may be disorient surgical vision and prevent complete decompression. However, there has been reported no evidence to suggest differences in rates of dural tears and CSF leak between MEL and conventional laminectomy in the meta-analysis [19]. In our study, the MEL group demonstrated a nonsignificant rate of dural tears and epidural hematoma relative to the SPSL group.

Both groups showed significant improvement in the JOABPEQ and VAS scores at 12 months after the primary surgery, but there was no significant difference between the two groups. MEL for lumbar spinal stenosis has been shown to demonstrate better scores in the JOABPEQ than those before surgery [20,21], which were similar to those in this study. Liu et al. [22] reported that the postoperative VAS score for low back pain was significantly lower with MEL than conventional laminectomy (1.0 versus 2.6, respectively). On the other hand, Rajasekaran et al. [23] reported no significant difference in the VAS score for back pain and neurogenic claudication between the SPSL group and conventional laminectomy group. Overdevest et al. [24] performed a systematic review and found that perceived recovery with techniques that preserve the posterior midline structures was generally equal to conventional laminectomy. There have been developed several patient-reported outcomes such as the Oswestry Disability Index, Roland-Morris Disability Questionnaire, or 36-Item Short-Form Health Surveys and JOABPEQ and VAS score assesses functional disability, perceived recovery, and pain. Ogura et al. [25] reported that the postoperative JOABPEQ score had a stronger correlation with patient satisfaction than the numerical rating scale, Roland-Morris Disability Questionnaire, and 8-Item Short Form Health Survey decompression surgery for lumbar spinal stenosis. It remains unclear which outcome measures can accurately reflect patient satisfaction in patients undergoing lumbar spinal surgery, and therefore further studies are necessary to clarify this issue.

This study has several limitations. The primary limitation is that the sample is small, and the difference between the sample size of each group is high. This study included only the patients who underwent posterior decompression for multi-segmental lumbar spinal stenosis because of a limited number of patients. Moreover, this is a retrospective study, so further prospective comparative studies of patients with these backgrounds may help determine which technique is more effective for posterior decompression for lumbar spinal stenosis.

## Conclusions

This study was retrospectively performed to compare the perioperative and postoperative outcomes of patients who underwent posterior decompression for multi-segmental lumbar spinal stenosis using MEL versus SPSL. This study suggests that both techniques are effective in treating multi-segmental lumbar spinal stenosis. There was no significant difference between the two procedures in IBL, complications rate, reoperation rate, or improvement in VAS and JOABPEQ scores. MEL had an advantage in the postoperative hospital stay.
